# Small-Molecule Modulation of the Circadian Clock in Cancer and Aging

**DOI:** 10.3390/molecules31091543

**Published:** 2026-05-06

**Authors:** Ashraf N. Abdo, Moustafa Gabr

**Affiliations:** Department of Radiology, Molecular Imaging Innovations Institute (MI3), Weill Cornell Medicine, New York, NY 10065, USA; asa4045@med.cornell.edu

**Keywords:** circadian rhythms, drug discovery, cancer therapeutics, aging

## Abstract

Circadian rhythms are ~24 h cycles regulated by an internal molecular clock. Disruption of this timing system has been implicated in numerous diseases, including the advancement of cancers and declines in function associated with aging. In recent years, scientists have identified various small molecules that can modulate core circadian clock proteins and pathways, providing potential therapeutic strategies to correct circadian dysfunction. This review presents a thorough overview of circadian clock mechanisms and highlights known small-molecule modulators. We describe how these compounds were discovered (through high-throughput screening and rational design) and categorize them based on their molecular targets. This review also summarizes key findings in cancer models, where clock-modulating compounds affect tumor metabolism, cell proliferation, and responsiveness to treatments, as well as in aging models, where strengthening circadian function may enhance metabolic health and longevity. Additionally, we cover clinical and preclinical studies involving these molecules and address challenges such as off-target effects and the complex nature of clock regulation. Finally, we outline future directions, emphasizing the development of new chronotherapeutics and the incorporation of circadian modulation into interventions for cancer and aging.

## 1. Introduction

Biological timing is essential for maintaining health and homeostasis. The circadian clock is an internal timekeeping system found in nearly every cell, generating roughly 24 h cycles in gene expression and physiology [1,2]. This system enables organisms to anticipate regular environmental changes (like daily light–dark cycles) and align their behaviors and metabolism accordingly. In mammals, timing is coordinated by a master pacemaker located in the brain’s suprachiasmatic nucleus (SCN), which synchronizes the clocks in peripheral organs throughout the body [3,4]. However, when circadian rhythms are persistently disrupted or thrown out of alignment due to factors such as shift work, frequent jet lag or social jet lag, or genetic defects in clock genes, a wide range of health problems can result [5]. Clock dysfunction has also been linked to metabolic diseases, mood disorders, and accelerated aging processes [6]. These observations suggest that pharmacologically adjusting the circadian system might provide therapeutic benefits.

Historically, medical approaches utilizing circadian biology have centered on chronotherapy—timing the delivery of existing medications to the body’s internal clock in order to maximize their effectiveness and minimize side effects [7]. For example, scheduling chemotherapy doses at times when healthy tissues are least susceptible to damage has shown success [8]. More recently, however, developing small-molecule drugs that specifically target components of the circadian clock itself has gained traction. In the last few years, scientists have discovered numerous compounds capable of resetting the clock’s phase, lengthening or shortening its period, or strengthening the amplitude of clock gene oscillations. By acting on core clock proteins or their regulatory pathways, these compounds represent a novel class of drugs often called clock modulators that could potentially manipulate circadian rhythms. Given that circadian disruption is linked to cancer and aging [6,9], researchers are exploring whether such clock modulators can suppress tumor growth, improve the outcomes of cancer therapies, or counteract physiological decline associated with aging. This review summarizes the molecular architecture of the circadian clock, highlights key small-molecule modulators and their discovery strategies, and critically evaluates evidence supporting their therapeutic potential in cancer and aging. We also discuss current clinical and preclinical studies, ongoing challenges, and future directions in this emerging field of chronopharmacology.

## 2. Molecular Mechanisms of Circadian Regulation

**Figure 1:**
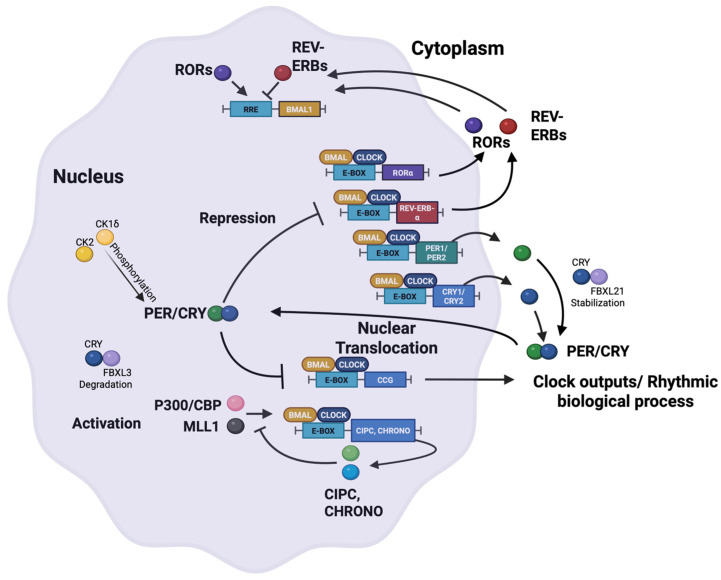
Molecular feedback loop of the mammalian circadian clock. The core transcriptional–translational feedback loops that drive ~24 h circadian rhythms in mammalian cells. In the nucleus, the BMAL1–CLOCK heterodimer binds E-box elements to activate expression of the core clock genes Per1/2, Cry1/2, Rev-erbα/β, and Rorα/β/γ, as well as downstream clock-controlled genes (CCGs). PER and CRY proteins accumulate in the cytoplasm, assemble into repressive complexes, and subsequently translocate back to the nucleus, where they inhibit their own transcription by inhibiting BMAL1–CLOCK activity and close the primary negative feedback loop. The stability and timing of PER and CRY proteins are regulated by post-translational modifications, particularly phosphorylation by casein kinases (CK1δ/ε and CK2), which influence nuclear entry and degradation dynamics. In addition, F-box proteins FBXL3 and FBXL21 control CRY protein turnover through ubiquitin-mediated proteasomal degradation, thereby fine-tuning circadian period and amplitude. A secondary loop is formed by REV-ERBs and RORs, which compete for ROR response elements (RREs) in the Bmal1 promoter to repress or activate transcription, respectively. BMAL1–CLOCK-driven transcription is further shaped by chromatin-modifying coactivators, including p300/CBP and MLL1, which enhance transcription through histone acetylation and methylation. Additional clock-associated repressors, such as CIPC and CHRONO, interact with the BMAL1–CLOCK complex to fine-tune transcriptional strength and rhythmic amplitude. Together, these interconnected regulatory layers generate robust circadian oscillations that control rhythmic physiological and biological outputs.

The mammalian circadian clock operates through a network of transcriptional–translational feedback loops in individual cells (Figure 1). At its core, a primary feedback loop is driven by BMAL1 and CLOCK, which are basic helix–loop–helix (bHLH) PAS-domain transcription factors that dimerize and bind to E-box sequences in DNA [10]. This BMAL1:CLOCK complex initiates rhythmic transcription of target genes, most prominently the period (Per1, Per2, Per3) and cryptochrome (Cry1, Cry2) genes [1,2,11]. After a delay, the encoded PER and CRY proteins accumulate in the cell (PER proteins are concurrently phosphorylated by casein kinase 1δ/ε) and assemble into PER–CRY complexes. These complexes translocate into the nucleus and inhibit the activity of the BMAL1:CLOCK complex [12]. This inhibition represses further transcription of the Per and Cry genes. As time passes, the PER and CRY proteins are gradually degraded via the ubiquitin–proteasome system (involving factors like β-TrCP targeting PER and the F-box protein FBXL3 targeting CRY) [13]. The degradation of PER and CRY lifts their inhibition on BMAL1:CLOCK, allowing a new cycle of gene transcription to begin. This entire negative feedback cycle takes roughly 24 h to complete, generating the daily oscillations in gene expression characteristic of circadian rhythms.

In parallel to the core loop, additional feedback loops enhance the stability and tune the period of the clock. One important secondary loop involves the nuclear receptors REV-ERBα/β (gene name *NR1D1/2*) and RORα/β/γ [1,2]. The BMAL1:CLOCK complex drives the expression of Rev-Erbα and Rev-Erbβ, and the resulting REV-ERB proteins act as strong repressors of Bmal1 transcription by competing with ROR proteins at ROR response elements (ROREs) on the Bmal1 promoter [14]. In contrast, RORs function as activators of Bmal1 expression. Together, the opposing effects of REV-ERBs (repressing) and RORs (activating) establish a reinforcing loop that helps maintain robust rhythmic expression of Bmal1 and many other clock-controlled genes. Another auxiliary loop includes transcription factors such as DBP and E4BP4, which themselves are regulated by the core clock and in turn control rhythmic expression of downstream genes (for example, Dbp and Nfil3/E4bp4 form a feedback circuit via D-box promoter elements) [14,15].

Another auxiliary feedback loop consists of repressors such as CIPC (CLOCK-interacting protein, circadian) [16] and CHRONO (CIART) [17], which form additional layers of regulation on the core CLOCK–BMAL1 complex. CIPC binds directly to CLOCK and inhibits its transactivation activity without disrupting its DNA binding, establishing a negative feedback loop that shapes the period and amplitude of circadian oscillations. CHRONO functions by associating with the C-terminal transactivation domain of BMAL1 and recruiting histone deacetylase HDAC1 to suppress E-box-mediated transcription, providing a parallel repressive arm alongside CRY proteins [17,18,19]. These repressors act in opposition to transcriptional coactivators such as CBP/p300 [20], which are histone acetyltransferases recruited by BMAL1:CLOCK to promote chromatin accessibility and rhythmic transcription [21]. MLL1 (mixed-lineage leukemia 1), a histone H3K4 methyltransferase, also plays a pivotal role by methylating circadian gene promoters, thus priming them for BMAL1:CLOCK activation and maintaining transcriptional rhythmicity [22]. The interplay between activators like CBP/p300 and MLL1 and repressors like CHRONO and CIPC exemplifies the multilayered epigenetic and transcriptional feedback architecture that further sustains robust circadian rhythms.

Beyond transcriptional loops, post-translational modifications of clock proteins are critical to precise timekeeping. For instance, casein kinase I (CK1) phosphorylation of PER1/2 promotes their degradation, effectively speeding up the clock [23], whereas AMP-activated protein kinase (AMPK) can phosphorylate CRY1 [24], tagging it for degradation through the E3 ubiquitin ligase FBXL3 [25]. Conversely, another E3 ligase, FBXL21, counterbalances FBXL3 in certain cellular compartments by stabilizing CRY proteins during part of the cycle [25]. Additionally, clock components undergo rhythmic acetylation and deacetylation modifications; for example, the deacetylase SIRT1 removes acetyl groups from BMAL1 and PER2 in an NAD^+^-dependent manner, linking energy metabolism to clock function [26]. Collectively, these integrated feedback loops and post-translational mechanisms produce a self-sustaining oscillator that is both accurate (keeping roughly 24 h periodicity) and adaptable (able to adjust timing in response to environmental cues).

The circadian system in mammals is organized in a hierarchical manner: each cell’s local clock is normally synchronized by the master pacemaker in the SCN of the brain. The SCN receives direct input from the eyes (retinal light signals) and uses the daily light/dark cycle to reset its phase every day [3]. The SCN then emits neural and hormonal signals that align the peripheral clocks throughout the body; for example, it drives daily fluctuations in hormones such as cortisol and melatonin, which in turn help coordinate rhythms in various organs [27]. Under typical conditions, this multi-level synchronization keeps tissue-specific rhythms in step with one another and with the external day–night cycle. Notably, circadian regulation is pervasive: estimates suggest that roughly 40% of all mammalian genes exhibit some degree of rhythmic expression in at least one organ [28]. These oscillating genes (often termed clock-controlled genes, or CCGs) govern a wide array of biological processes, from cell division and DNA repair to metabolism and immune responses. If any part of this clock network is disrupted, whether by genetic mutations, the aging process, or environmental factors, rhythmic gene expression can become disrupted, leading to various physiological dysfunctions. As we explore in the next sections, small-molecule compounds provide a way to pharmacologically tweak the clock’s mechanism, to either fix abnormal rhythms or intentionally shift the timing system for therapeutic purposes.

While the core molecular clock mechanism is broadly conserved across tissues, its downstream outputs are highly organ-specific, reflecting the unique physiological functions of each tissue. In the liver, circadian rhythms play an important role in regulating several critical metabolic processes, such as gluconeogenesis, lipid synthesis, and detoxification of xenobiotic substances via circadian rhythmicity in the expression of genes such as Pck1, Srebp1c, and cytochrome P450 enzymes. Disruption of the hepatic clock has been shown to lead to metabolic dysfunction (i.e., hepatic steatosis and insulin resistance), emphasizing the importance of the hepatic circadian clock in the maintenance of metabolic homeostasis [6,28].

Circadian rhythms also regulate mitochondrial oxidative capacity, glucose uptake by skeletal muscle, and muscle performance. Disruption of the muscle-specific clock impairs insulin sensitivity and reduces metabolic flexibility in skeletal muscle, at least in part, due to altered expression of genes associated with mitochondrial function and energy metabolism [29,30].

Circadian regulation of lipid storage, endocrine signaling, and thermogenic responses occurs in the adipose tissue. Recent studies have demonstrated that disruption of circadian alignment results in altered metabolic programming of brown adipose tissue (BAT), leading to impaired thermogenesis and decreases in energy expenditure. Restoration of circadian alignment, using either behavioral or molecular approaches, results in re-establishment of rhythmic gene expression and metabolic function in BAT, thus supporting the idea that circadian disruption of metabolic function is reversible [31].

These findings are directly relevant to the concept of organ-specific aging, in which different tissues exhibit distinct patterns of circadian decline over time. Aging is associated with reduced amplitude, phase instability, and impaired re-entrainment of circadian rhythms; however, these effects are not uniform across organs [32]. Metabolic tissues such as liver and adipose tissue often show earlier and more pronounced circadian disruption, contributing to age-associated metabolic diseases, whereas other tissues may retain partial rhythmicity. Recent studies further demonstrate that circadian misalignment under metabolic stress can drive widespread reprogramming of tissue-specific transcriptional and metabolic networks, leading to loss of temporal coordination in energy metabolism and cellular function, while restoration of circadian alignment can partially reverse these alterations and re-establish rhythmic gene expression programs [32]. Importantly, organ-specific aging reflects not only differences in the magnitude of circadian decline but also divergence in downstream functional consequences, as circadian dysregulation impacts tissue-specific processes such as metabolism, repair capacity, and cellular stress responses. This differential vulnerability suggests that aging progresses through asynchronous deterioration across organ systems, rather than as a uniformly coordinated process. Consequently, circadian disruption may act as both a driver and amplifier of tissue-specific aging phenotypes, reinforcing the need for therapeutic strategies that account for organ-level circadian dynamics. This heterogeneity supports the view that aging is not a uniform systemic process but rather a mosaic of tissue-specific functional decline, with circadian dysregulation acting as a central coordinating factor [9,33].

These examples highlight how circadian regulation extends beyond a centralized timing system to orchestrate tissue-specific physiological processes, underscoring the potential for targeted circadian modulation to produce organ-specific therapeutic benefits.

## 3. Small-Molecule Modulators of Circadian Rhythms

### 3.1. Discovery Strategies

Researchers have employed both unbiased high-throughput screening and rational design to identify circadian rhythm modulators. One major strategy is phenotypic high-throughput screening using cellular clock reporter systems. In these assays, human or rodent cells are engineered with a bioluminescent or fluorescent reporter under the control of a core clock gene promoter (for example, a Bmal1-luciferase or Per2-luciferase construct) [34,35]. Large chemical libraries are applied to these reporter cells, and automated monitoring of the cells’ oscillation parameters (period, phase, amplitude of the luminescence rhythms) is used to detect “hits”, compounds that significantly alter circadian timing. This approach has led to the discovery of several first-in-class clock modulators. For instance, Hirota et al. screened approximately 120,000 compounds and identified Longdaysin, a small molecule that dramatically extended the circadian period in human cells and even in SCN tissue explants [36]. This study revealed that Longdaysin achieves this effect by simultaneously inhibiting multiple kinases involved in clock protein turnover, namely CK1δ/ε, CK1α, and ERK2. In another landmark screen, a library of 60,000 compounds was tested for period length effects, yielding the carbazole derivative KL001 as a hit that lengthened the period of cellular rhythms [37]. Biochemical pull-down experiments determined that KL001 targets the cryptochrome proteins CRY1 and CRY2. KL001 binds in the flavin adenine dinucleotide (FAD) pocket of CRY, thereby preventing the ubiquitin ligase FBXL3 from recognizing and degrading CRY. By stabilizing CRY proteins, KL001 effectively slows the clock (lengthening the cycle length) and also alters the amplitude of clock gene oscillations [37]. These examples demonstrate the power of unbiased screening to uncover clock modulators with novel mechanisms of action.

Rational, target-driven approaches have also been productive, focusing on clock components with known ligand-binding sites or enzymatic activity. Several circadian proteins are inherently “druggable”, for example, the ligand-binding domains of the nuclear receptors REV-ERB and ROR, or the active sites of kinases like CK1 and CK2 that modify clock proteins. Researchers have deliberately targeted these components. For instance, the orphan nuclear receptors REV-ERBα and REV-ERBβ (which naturally bind heme) were identified as appealing targets to chemically inhibit the clock’s positive arm (since activating REV-ERB effectively represses Bmal1 transcription). The first synthetic REV-ERB agonist, GSK4112 (also called SR6452) [38], was obtained through a combination of rational design and library screening; GSK4112 itself had only moderate potency and pharmacokinetic properties, but its chemical scaffold provided a starting point for developing more potent analogs, such as SR9009 and SR9011 [39]. Likewise, structure-based drug design was applied to the RORα/γ receptors: this led to the development of SR1078, a synthetic agonist of RORα/γ that can upregulate ROR target genes [40].

Target-centric screens have also yielded compounds that modulate circadian-relevant kinases. A prominent example is GO289 [41], a potent and selective inhibitor of casein kinase 2 (CK2), which was discovered using a combination of cell-based screening and chemoproteomics. CK2 was not originally recognized as a core clock regulator, but the effects of GO289 revealed that blocking CK2-mediated phosphorylation on clock proteins could significantly lengthen the circadian period. Intriguingly, GO289 also demonstrated anti-cancer activity (discussed later), linking the clock to cell growth pathways [41].

Besides novel compound discovery, researchers have explored repurposing known drugs and using in silico modeling to find clock modulators. Retrospective analyses of existing drug libraries have revealed that many clinically used drugs can incidentally influence circadian parameters. In one such study, roughly 5% of tested compounds including various kinase inhibitors and chemotherapeutic agents were found to alter the circadian period in cell-based assays [42]. For example, certain FDA-approved inhibitors of CDKs, GSK3, and JNK kinases, as well as some microtubule-targeting drugs, were observed to lengthen or shorten the clock period, likely by acting on common cellular signaling nodes [42]. These observations suggest that some existing medications could potentially be repurposed as chronotherapeutics or at least serve as a starting point for designing new derivatives that more specifically target circadian pathways.

Finally, structure-guided design, supported by crystallography of clock protein complexes, is beginning to reveal druggable pockets on core circadian proteins themselves. For instance, detailed knowledge of the interaction interface between CRY and FBXL3 [43], as well as the PAS-domain structure of the CLOCK:BMAL1 complex [1], has informed efforts to create molecules that disrupt these protein–protein interactions.

In summary, through a combination of unbiased high-throughput screens, focused ligand design, repurposing of known drugs, and rational structure-based approaches, researchers have assembled a growing toolkit of small molecules capable of fine-tuning the circadian clock.

### 3.2. Classification of Circadian Clock Modulators

**Figure 2:**
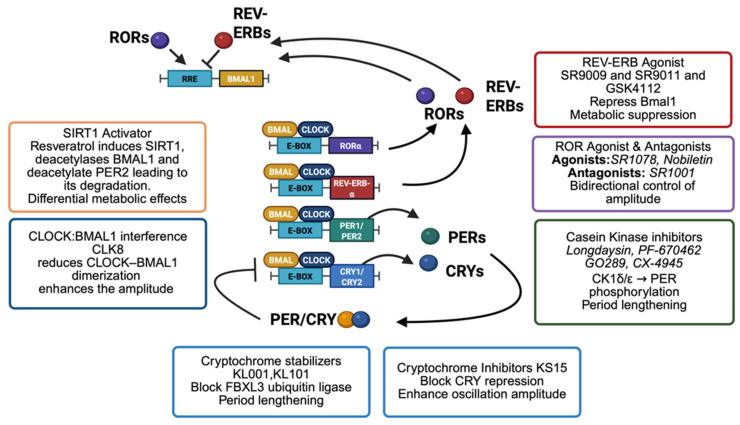
Classification of small-molecule circadian clock modulators. This figure outlines the main classes of small molecules that regulate the mammalian circadian clock, grouped by their molecular targets and functional effects. Cryptochrome (CRY) stabilizers, such as KL001 and KL101, protect CRY proteins from degradation, extending the repressive phase and lengthening the circadian period. In contrast, CRY inhibitors (e.g., KS15) weaken CRY-mediated repression of the BMAL1:CLOCK complex, increasing the amplitude of clock gene oscillations with little effect on period length. REV-ERB agonists (SR9009, SR9011, GSK4112) enhance transcriptional repression of *Bmal1* and dampen clock-controlled metabolic programs, whereas ROR modulators exert bidirectional control: ROR agonists (SR1078, nobiletin) strengthen rhythmic gene expression, while ROR inverse agonists (SR1001) suppress *Bmal1*. Casein kinase inhibitors targeting CK1δ/ε or CK2 (Longdaysin, PF-670462, GO289, CX-4945) alter PER phosphorylation and stability, producing marked changes in circadian period. Sirtuin activators, such as resveratrol, link clock regulation to cellular metabolism.

Small-molecule clock modulators can be categorized by their molecular targets or modes of action. Below we highlight major classes of these compounds and representative examples (Figure 2):

- Cryptochrome Stabilizers: These molecules bind to CRY1 and CRY2, protecting them from ubiquitination and subsequent degradation. By stabilizing CRY, they prolong the repressive phase of the clock cycle. Example: KL001 and its analogs (such as KL101) bind within the FAD-binding pocket of cryptochrome, which prevents the FBXL3 ubiquitin ligase from docking [37]. As a result, CRY proteins accumulate, leading to a lengthened circadian period. In diabetic mouse models, treatment with a KL001 derivative improved glucose homeostasis, demonstrating the therapeutic potential of clock modulation through CRY stabilization.
- Cryptochrome Inhibitors: These compounds reduce or block the repressive function of CRY proteins, thereby effectively “releasing the brake” on the clock and enhancing its output. Example: KS15 is a synthetic small molecule discovered in a chemical library screen that binds to CRY1/2 (without binding BMAL1 or CLOCK) and impairs CRY’s ability to inhibit the BMAL1:CLOCK transcriptional complex. By doing so, KS15 lifts the repression on clock-controlled genes, increasing their expression [44]. In particular, KS15 was reported to elevate levels of the clock output gene Per2, which in turn led to anti-proliferative and pro-apoptotic effects in cancer cell lines. Unlike KL001 (a CRY stabilizer that lengthens the circadian period), KS15 primarily boosts the amplitude of clock gene oscillations and does not substantially change the period length. Another example, M47, selectively binds to and destabilizes the core clock protein CRY1, increasing its ubiquitination and degradation, lengthening the cellular circadian period, enhancing apoptosis in p53-deficient cells, and extending the lifespan of p53−/− mice, demonstrating CRY1 destabilization as a promising strategy for targeting circadian regulation in disease contexts [45].
- REV-ERB Agonists: These compounds activate the nuclear receptors REV-ERBα and REV-ERBβ, which function as transcriptional repressors of Bmal1 and other clock genes, effectively damping the positive limb of the clock. Examples: SR9009 and SR9011 are potent synthetic REV-ERB agonists that reduce Bmal1 expression and, at sufficiently high concentrations, can even eliminate detectable circadian oscillations. By locking REV-ERB in its active (repressive) state, these drugs suppress the transcription of numerous metabolic genes [39]. In cell studies, SR9009 and SR9011 were shown to decrease lipid synthesis and alter glucose metabolism [46]. An earlier prototype REV-ERB agonist, GSK4112, provided initial proof-of-concept but had some off-target effects (for example, unintended activation of LXR); subsequent analogs (such as those in the GSK2945 series) have been developed with improved specificity for REV-ERBs [47].
- ROR Agonists and Antagonists: The ROR (retinoic acid-related orphan receptor) family (RORα/β/γ) acts in opposition to REV-ERBs by promoting Bmal1 transcription, so modulating ROR activity can either enhance or diminish clock gene expression. Examples: SR1078 is a synthetic agonist of RORα/γ that boosts the expression of ROR-regulated genes. In cancer cell studies, SR1078 treatment stabilized the tumor suppressor p53 and triggered apoptosis, suggesting that it can influence both circadian targets and tumor suppressor pathways [40]. On the other hand, SR1001 is a RORα/γ inverse agonist (functional antagonist) that inhibits ROR activity, producing effects similar to activating REV-ERB (i.e., repression of Bmal1) [48]. Notably, the natural compound (derived from citrus peel) was recently identified as a ROR agonist that strengthens circadian rhythms in cells. Nobiletin binds to RORs with high affinity and modestly increases the expression of ROR target genes (including Bmal1), thereby enhancing the amplitude of oscillations in clock and metabolic gene expression [49]. Interestingly, both activating ROR and inhibiting ROR have yielded beneficial outcomes in different metabolic disease models, indicating that the therapeutic effect may depend on physiological context and specific downstream pathways.
- Casein Kinase Inhibitors: Blocking kinases that phosphorylate core clock proteins can significantly change the clock’s speed (period length). The casein kinase family is a key example: CK1δ and CK1ε normally phosphorylate PER proteins to promote their degradation [12], which helps determine the circadian period length. Examples: Longdaysin (discussed earlier) inhibits CK1δ/ε (as well as CK1α and ERK2), producing a dose-dependent extension of the circadian period by several hours beyond normal [36]. Selective CK1δ/ε inhibitors, such as PF-670462, likewise lengthen the period and have been used in animal studies for clock resetting experiments [50]. Inhibiting casein kinase 2 (CK2) has emerged as a new strategy: GO289 is a highly potent and selective CK2 inhibitor that interferes with multiple phosphorylation events in the clock, leading to a marked lengthening of the circadian period. GO289 was particularly illuminating because it identified CK2 as a critical link between the circadian clock and cell growth signaling; treatment with GO289 not only altered circadian rhythms but also reduced cancer cell proliferation (as discussed later) [41]. Another CK2 inhibitor, CX-4945 (Silmitasertib), which is already in use as an anti-cancer drug, has been noted to affect circadian timing as well, though it is less selective and hits additional kinase targets [51].
- Sirtuin Activators: SIRT1 is an NAD^+^-dependent deacetylase that interacts with core clock machinery by deacetylating proteins like BMAL1 and PER2, thereby linking circadian regulation to cellular metabolic state [26]. Enhancing SIRT1 activity can bolster certain aspects of clock function. Example: Resveratrol, a plant-derived polyphenol known to activate SIRT1, has been shown not only to extend lifespan in some model organisms but also to alter circadian gene expression patterns and strengthen behavioral rhythms. Although resveratrol affects multiple pathways (it is a pleiotropic compound), its known impact on the circadian system potentially through raising cellular NAD^+^ levels and activating SIRT1 indicates that sirtuin activators in general may modulate clock outputs in ways that could be beneficial for aging and longevity [52].
- CLOCK:BMAL1 Interference: Scientists identified a novel small molecule, CLK8, that disrupts the interaction between CLOCK and its partner BMAL1 [53]. Using structure-based virtual screening followed by biochemical and cell-based assays, the authors showed that CLK8 reduces CLOCK–BMAL1 dimerization and interferes with CLOCK’s nuclear translocation both in vitro and in vivo. This targeted perturbation of the positive arm of the transcription–translation feedback loop leads to relative dominance of the negative arm and enhances the amplitude of circadian rhythms in U2OS cells without significantly altering period length. CLK8 thus acts as a molecular tool to selectively modulate circadian rhythm amplitude and highlights CLOCK–BMAL1 interactions as a pharmacological target for therapies aimed at disorders involving dampened circadian rhythms [53].

In summary, studies have shown a broad range of small molecules capable of accelerating or decelerating the biological clock, strengthening its oscillation amplitude, or shifting its phase by acting on various clock components. The capability to pharmacologically fine-tune the circadian system with such precision has opened new avenues for research both to deepen our understanding of clock biology and to develop therapies for disorders linked to circadian dysregulation. In the next sections, we explore how these clock-modulating compounds perform in the contexts of cancer and aging, two areas where disruptions of circadian timing have particularly significant consequences.

### 3.3. Evidence in Cancer Models

Circadian clock disruption can strongly influence cell cycle regulation, DNA damage repair, and metabolic pathways, processes intimately involved in cancer biology [54]. Many tumors exhibit altered or dampened expression of circadian genes. In some cancer models, core clock genes appear to maintain largely intact circadian period and amplitude with a change in circadian phase, whereas clock-controlled genes display remarkable dysregulation [54]. Notably, many of these clock-controlled output genes regulate key metabolic pathways, and their disruption contributes to the metabolic reprogramming that supports cancer cell growth and survival. Such findings have prompted the hypothesis that restoring or adjusting circadian clock function in cancer cells could suppress tumor progression or improve responses to cancer treatment by re-aligning tumor metabolism with normal circadian timing. Small-molecule clock modulators are invaluable tools to test this idea in preclinical settings. Indeed, a growing body of evidence indicates that pharmacologically targeting the circadian clock can slow down cancer cell proliferation, promote apoptosis (programmed cell death), and enhance the efficacy of conventional anti-cancer treatments. A summary of key small-molecule circadian modulators evaluated in cancer models, including their molecular targets, mechanisms of action, and observed anti-tumor effects, is provided in Table 1.

One line of evidence comes from compounds that target cryptochrome. As a core repressor in the clock, CRY has been found to support the viability of certain cancer cells. Using a synthetic CRY inhibitor like KS15 (described above) can lift CRY-mediated repression, leading to increased expression of clock-controlled tumor suppressor genes such as PER2. In estrogen receptor-positive breast cancer cells (MCF-7), treatment with KS15 was shown to elevate PER2 levels and produce anti-proliferative effects, including an increase in apoptosis [44,55]. Notably, KS15 also made these cancer cells more vulnerable to standard chemotherapeutic agents: co-treatment with KS15 enhanced the cell-killing effects of both doxorubicin and tamoxifen in MCF-7 cultures [44]. This finding suggests that clock modulators could potentially be used to sensitize tumors to existing chemotherapy, perhaps by pushing cancer cells into a less proliferative and more drug-sensitive state through regulation of the circadian machinery. In line with this, a prior study reported that a different CRY inhibitor (described by Chun et al. in 2015) slowed the growth of breast cancer cells and increased their sensitivity to the chemotherapeutic drug oxaliplatin, further supporting CRY as a promising oncologic drug target [55]. Similar data was shown by Gul et al., showing that M47 enhanced tumor apoptosis in p53-deficient cells, and extended the lifespan of p53−/− mice by destabilizing CRY1 by increasing its ubiquitination and degradation [45].

Among clock-targeting compounds, REV-ERB agonists have demonstrated particularly robust anti-tumor activity across multiple cancer models. By activating REV-ERBα/β, these compounds suppress Bmal1 and numerous metabolic genes, thereby crippling a cancer cell’s metabolic capacity. In vitro experiments showed that the REV-ERB agonist SR9009 was lethal to a wide variety of cancer cell types, including leukemia, breast, colon, and melanoma cell lines [39]. Cancer cells treated with SR9009 exhibited greatly reduced viability accompanied by a decline in oxidative metabolism (evidenced by lower reactive oxygen species production and impaired lipid utilization). In synchronized cell populations, SR9009 also caused the majority of cells to arrest in the G0/G1 phase of the cell cycle. Crucially, these anti-tumor effects have translated into animal models: In mice bearing human small-cell lung carcinoma (SCLC) xenografts, SR9009 administration significantly suppressed tumor growth in vivo. Mechanistic studies in the SCLC context traced SR9009’s cytotoxicity to an inhibition of autophagy [56]. SR9009 enhanced REV-ERB’s repression of the autophagy gene Atg5, thereby blocking autophagosome formation, while simultaneously triggering apoptosis through caspase-3 activation [56]. These multi-faceted actions like metabolic disruption, cell cycle arrest, and autophagy inhibition highlight how modulating the circadian clock can expose and exploit vulnerabilities in cancer cells. It is worth noting, however, that SR9009 may exert certain anti-proliferative and metabolic effects even in the absence of REV-ERB. This suggests caution in interpreting its mechanism and underscores the importance of considering potential off-target effects [57].

**Table 1:** Small-molecule circadian clock modulators evaluated in cancer models. Overview of circadian clock-targeting compounds and genetic interventions evaluated in cancer models, highlighting their molecular targets, mechanisms of action, and observed anti-tumor effects.

Target/Pathway	Compound/Approach	Cancer Model	Primary Mechanism	Observed Effects	Key Implications	Chemical Structure	Key Reference
Cryptochrome (CRY)	KS15	MCF-7 breast cancer cells	Inhibits CRY repression; increases PER2	Anti-proliferative, increased apoptosis; sensitizes cells to doxorubicin and tamoxifen	CRY inhibition sensitizes tumors to chemotherapy	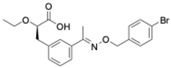	Chun et al., Biochem Biophys Res Commun, 2015 [44,55]
Cryptochrome (CRY1)	M47	p53-deficient cancer cells; p53−/− mice	Promotes CRY1 ubiquitination and degradation	Enhanced apoptosis; lifespan extension in p53−/− mice	CRY destabilization as oncologic strategy	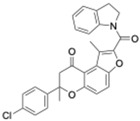	Gul et al., Nat Commun, 2022 [45]
REV-ERBα/β	SR9009 and its analogs	Leukemia, breast, colon, and melanoma cells; SCLC xenografts	Represses Bmal1 and metabolic genes; inhibits autophagy	Cell cycle arrest, metabolic collapse, apoptosis; tumor growth suppression in vivo	Metabolic vulnerability via circadian repression	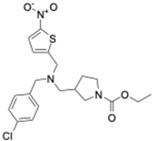	Sulli et al., Nature, 2018 [39]
RORα/γ	SR1078	HepG2 liver cancer cells	ROR activation stabilizes p53	Growth arrest and apoptosis	ROR agonism reactivates tumor suppressor pathways	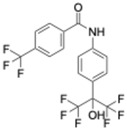	Wang et al., ACS Chem Biol, 2010 [40]
REV-ERBβ/autophagy	ARN5187	Breast cancer cell lines	Blocks autophagosome maturation	Reduced viability; selective tumor cytotoxicity	Dual clock–autophagy targeting	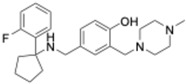	De Mei et al., Oncogene, 2015 [58]
CK2 (clock-associated kinase)	GO289	Cancer cell lines; mouse tumor models	Inhibits CK2 phosphorylation of clock proteins	Reduced proliferation; slowed tumor progression	Kinase–clock axis suppresses tumor growth	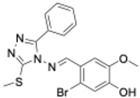	Oshima et al., Sci Adv, 2019 [41]
Genetic clock modulation	PER2 overexpression; BMAL1 knockdown	Pancreatic and other cancer cell lines	Restores circadian tumor-suppressive signaling	Reduced growth; increased chemosensitivity	Circadian reactivation is tumor-suppressive		Fu et al., Cell, 2002 [59]

Modulating the ROR nuclear receptors has also shown encouraging results in cancer models. RORα expression is often downregulated in certain cancers (such as breast, ovarian, and prostate cancers), and stimulating ROR signaling in these settings can lead to growth arrest of the cancer cells. For example, the RORα/γ agonist SR1078 was found to stabilize the tumor suppressor protein p53 and induce apoptosis in HepG2 liver cancer cells [40]. Conversely, inhibiting ROR activity (which has a similar net effect to activating REV-ERB) has likewise produced anti-cancer effects. A notable case is ARN5187, a compound identified as a REV-ERBβ antagonist that also functions as an autophagy inhibitor [58]. In breast cancer cells, ARN5187 impaired the maturation of autophagosomes (analogous to the effect of chloroquine, but more potent) and reduced cell viability, particularly in cells with high REV-ERBβ levels. Optimized analogs of ARN5187 demonstrated broad cytotoxic effects across various tumor cell lines, while sparing non-cancerous cells [58]. These findings highlight that both sides of the ROR/REV-ERB regulatory axis are druggable vulnerabilities in cancer cells; either activating ROR or hyper-activating REV-ERB can drive cancer cells toward apoptotic cell death, depending on the tumor context.

Another link between the circadian clock and cancer has been highlighted by compounds targeting clock-associated kinases, such as GO289. GO289’s main target, CK2, is a kinase frequently overexpressed in cancers that contributes to pro-proliferative signaling [41]. Inhibition of CK2 by GO289 within the context of the clock was found to both lengthen the circadian period and simultaneously hinder cancer cell growth. It has been shown that GO289 reduced the phosphorylation of multiple clock proteins, thereby dampening the amplitude of clock oscillations, and importantly, it also decreased the proliferation of cancer cells in vitro and slowed tumor progression in mouse models. This work provided a direct demonstration that circadian clock modulation can translate into tumor growth suppression [59]. The implication is that drugs targeting the clock might tackle cancer on dual fronts: by disrupting key growth-promoting pathways in tumor cells (in this case, via CK2 inhibition) and by reasserting circadian control over the cell cycle, potentially slowing the rampant cell division characteristic of cancer.

Genetic approaches have also been employed in tumor models to corroborate the effects of clock modulation observed with drugs. For instance, experimentally increasing the expression of PER2 (a clock protein often found at low levels in tumors) in pancreatic cancer cells was shown to slow their growth and make them more susceptible to platinum-based chemotherapy [59,60]. Similarly, in some cancer cell lines, knocking down the core clock driver BMAL1 reduced cell viability or overcame resistance to certain drugs [61]. Interestingly, in other cancer contexts, BMAL1 loss can actually promote tumor growth, indicating that the role of specific clock components may vary by cancer type [62]. Taken together, these genetic studies support the general concept that re-establishing a robust circadian rhythm in cancer cells tends to be tumor-suppressive. From a therapeutic standpoint, small-molecule modulators provide a means to achieve this clock reactivation in a controlled and reversible way, without needing direct genetic alterations.

In summary, studies in cancer models suggest that circadian clock modulators can function as anti-cancer agents through multiple mechanisms: they can arrest cell cycle progression, reactivate apoptotic pathways, deprive cancer cells of metabolic resources, and defeat autophagy-based survival strategies. For example, REV-ERB activators such as SR9009 disrupt tumor metabolism and cell cycle regulation, driving cancer cells into apoptosis [39]; CRY inhibitors like KS15 increase the expression of clock-controlled tumor suppressor genes and have been shown to work synergistically with chemotherapy [55]; and kinase inhibitors like GO289 tap into circadian-related kinase pathways to suppress cancer cell growth [41].

While enhancing circadian rhythmicity has been associated with improved outcomes in cancer treatments, it is increasingly evident that suppressing certain aspects of the clock may offer therapeutic avenues in other settings, particularly in cancer. For example, pharmacological agents like those targeting BMAL1 or CLOCK attenuated mitochondrial metabolic function and reduced expression of tricarboxylic acid cycle enzymes. Small-molecule agonists of two independent BMAL1–CLOCK negative regulators, cryptochromes and REV-ERBs, downregulated stem cell factors and reduced glioblastoma stem cell growth [63]. These findings challenge the assumption that stronger oscillations universally signify better health and suggest that circadian modulation should be tailored to disease context. In some cases, reducing rhythmic output may disrupt pathological processes more effectively than restoring robust oscillations. This highlights the importance of a nuanced view in which either reinforcement or attenuation of circadian function may be appropriate, depending on the specific physiological or pathological target.

These encouraging findings provide a strong rationale for further development of clock-targeted drugs in oncology. It is conceivable that in the future, standard cancer therapy regimens might include a chronotherapeutic element, whether it be timing conventional drugs to a patient’s circadian cycle or adding a clock modulator to strengthen the body’s natural anti-tumor rhythms. While results from cell and animal models are promising, moving these strategies into clinical practice will require additional validation and carefully designed trials.

### 3.4. Evidence in Aging Models

**Figure 3:**
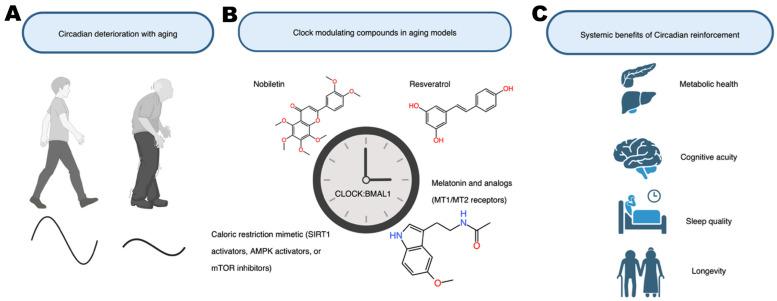
Circadian clock modulation as a strategy to promote healthy aging. This figure illustrates how aging weakens circadian rhythm amplitude and stability, and how small-molecule interventions targeting the circadian clock can counteract age-related physiological decline. (**A**) Aging is associated with reduced rhythm amplitude, impaired re-entrainment, and deterioration in metabolic, sleep, hormonal, and cognitive rhythms. (**B**) Several classes of circadian-active compounds improve rhythmic function in aging models: the ROR agonist nobiletin enhances circadian amplitude and metabolic rhythms; the SIRT1 activator resveratrol promotes BMAL1/PER2 deacetylation and restores youthful clock dynamics; CR mimetics (e.g., AMPK and mTOR pathway modulators) strengthen clock–metabolism coupling; and melatonin or its analogs reinforce SCN signaling through MT1/MT2 receptors and support circadian phase alignment. (**C**) Strengthening circadian rhythms through these interventions improves metabolic health (increased energy expenditure, improved insulin sensitivity, reduced hepatic fat), supports brain function and neuroprotection, enhances sleep quality, and may contribute to lifespan extension. Overall, the beneficial effects of these compounds depend on the presence of an intact circadian clock.

Aging is associated with a well-documented decline in the strength and stability of circadian rhythms [64]. Older adults (and aged rodents) typically show reduced amplitudes in many daily cycles; for example, elderly individuals often have weaker oscillations in core body temperature, sleep–wake activity patterns, nighttime melatonin secretion, and cortisol levels [9]. At the molecular level, aging is accompanied by altered expression of core clock genes in peripheral tissues and a slower ability to re-entrain to shifts in the light–dark cycle. This age-related breakdown of circadian regulation is believed to contribute to the deterioration of metabolic and cognitive functions over time, as well as to a higher incidence of chronic diseases. Conversely, evidence suggests that maintaining or enhancing robust circadian rhythms may support healthier aging [64]. Notably, exceptionally long-lived animal models tend to preserve strong circadian rhythms: for instance, a strain of transgenic mice (αMUPA mice) that live much longer than normal mice also retains high-amplitude circadian behavioral and gene expression rhythms even in old age, correlating with their extended lifespan [65]. On the flip side, disrupting core clock genes can accelerate aging-like symptoms: mice entirely lacking Bmal1 not only become arrhythmic but also exhibit premature aging phenotypes (such as muscle loss, cataract development, and shortened lifespan) [66]. These findings set the stage for interventions that target the circadian clock as a means to counteract certain aspects of the aging process (Figure 3).

Small-molecule clock modulators have been explored in various experimental models for their ability to combat age-related decline or even extend healthy lifespan. One major area of interest is metabolic aging, since conditions like metabolic syndrome and energy imbalance become more common with age and are associated with weakening circadian rhythms. The natural compound nobiletin offers a striking example of a clock-targeted intervention for metabolic health [49]. As mentioned earlier, nobiletin (NOB) is a citrus-derived flavonoid that enhances circadian rhythms by acting as a ROR agonist. When given to middle-aged obese mice, nobiletin effectively rejuvenated their metabolic rhythms and improved key metabolic health indicators. In both diet-induced obese mice and diabetic (db/db) mice, NOB treatment restored robust oscillations of core clock gene expression in the liver, increased the amplitude of daily locomotor activity rhythms, and markedly improved metabolic outcomes. Nobiletin-treated animals gained less weight (despite no reduction in food intake), showed higher energy expenditure and greater fatty acid oxidation, exhibited improved glucose tolerance and insulin sensitivity, and accumulated less fat in the liver. Strikingly, these metabolic benefits were largely absent in ClockΔ19/Δ19 mutant mice, indicating that an intact circadian clock was necessary for nobiletin’s effects [49]. This implies that nobiletin was exerting its anti-aging metabolic benefits through circadian mechanisms. Although that particular study did not measure lifespan directly, the improvements in metabolic health and the reduction in fatty liver suggest a potential for delaying age-associated metabolic disorders. Additionally, nobiletin has demonstrated neuroprotective properties in models of cognitive decline (for example, it improved memory performance in a mouse model of Alzheimer’s disease) [67], hinting that strengthening circadian function might also benefit aspects of brain aging.

Another interesting compound is resveratrol, which has been heralded for its lifespan-extending effects in several model organisms. Resveratrol is known to activate SIRT1, and through this action, it can influence the circadian clock in mammals [52]. Studies have found that resveratrol can increase the amplitude of certain clock gene oscillations and improve age-related physiological rhythms in mice. For instance, resveratrol treatment was reported to shift the phase of the liver’s clock and to adjust the rhythm of insulin release, effectively making these rhythms in older mice resemble those of younger animals.

Moreover, given that interventions like time-restricted feeding (a regimen where food intake is limited to the active phase of the day) and caloric restriction both strengthen circadian oscillations (for example, feeding only during an organism’s active period enhances the amplitude of clock gene rhythms in peripheral tissues) [68,69,70,71], it stands to reason that pharmacological mimics of these interventions might derive some of their beneficial effects by modulating circadian timing. Supporting this notion, a recent study demonstrated that simply aligning feeding times with the circadian cycle in mice (without reducing overall calorie intake) was sufficient to extend their lifespan by roughly 35% [70]. This striking result underscores the importance of circadian alignment for longevity and suggests that drugs which promote robust circadian rhythms could serve as longevity therapeutics in their own right.

Recent evidence has expanded our understanding of how behavioral interventions, such as time-restricted feeding (TRF), interact with peripheral circadian clocks. While not pharmacological in nature, TRF studies have shed light on the physiological consequences of restoring or reinforcing circadian rhythms and have informed mechanistic targets for small-molecule development. Notably, recent work by Hepler et al. (2026) demonstrated that TRF can restore disrupted circadian metabolic programming in brown adipose tissue (BAT) in the context of diet-induced obesity, effectively re-engaging thermogenic capacity and peripheral clock gene expression [31]. These findings emphasize that circadian alignment, even when achieved through nonpharmacological means, can recalibrate metabolic outputs and may mimic or enhance the action of clock-modulating compounds. Although discussed briefly here for conceptual relevance, this example underscores how rhythm-aligned interventions help contextualize the therapeutic logic behind targeting circadian networks.

Further linking circadian function to aging outcomes are findings related to sleep and cognition in the elderly. The hormone melatonin, which is governed by the central clock and in turn helps reinforce SCN rhythms, declines significantly with advanced age [9,72]. In some studies, nightly supplementation of melatonin has been used to improve sleep quality in older adults. Such treatment not only addresses insomnia symptoms but may also help amplify overall circadian rhythm strength [73]. Although melatonin does not directly bind core clock proteins, administering it in older people aims to restore a robust nocturnal signal, which could help synchronize and invigorate their peripheral clocks. Preclinical studies suggest that long-term melatonin supplementation may preserve youthful circadian hormone profiles and attenuate age-associated oxidative stress, although definitive evidence in humans remains limited [74]. However, rigorous human trials are needed to confirm any concrete benefits of melatonin (or other clock-targeted interventions) on aging biomarkers or on extending lifespan.

In summary, small molecules that bolster circadian function show significant promise for alleviating aging-related declines. By enhancing the amplitude of circadian rhythms or tweaking the timing of the clock, these compounds can potentially correct the “clock dysfunction” seen in older organisms. The example of nobiletin is particularly illustrative: it directly targets a core clock mechanism (ROR nuclear receptors) and produces wide-ranging physiological benefits in metabolic and even cognitive aspects of health [49]. Likewise, SIRT1 activators such as resveratrol, while less specific in action, indicate that manipulating clock-associated pathways can yield longevity-related benefit [52]. It is important to note that the relationship between circadian rhythms and aging is bidirectional: not only can a stronger circadian system promote healthier aging, but also known longevity interventions (like caloric restriction, regular exercise, or certain pharmaceuticals) often feed back to enhance circadian rhythm robustness [64,69,70]. Therefore, it is conceivable that future anti-aging treatment strategies will intentionally incorporate chronotherapeutic elements. That said, applying these findings to humans will require careful research, as human aging is complex and multifactorial. Still, the insights gained so far provide a strong rationale for moving forward with clinical exploration of clock modulators as a means to support healthy aging and to treat age-related disorders such as neurodegeneration, frailty, and metabolic syndrome.

## 4. Challenges and Limitations

Despite the enthusiasm for clock-targeted therapies, several significant challenges must be navigated as the field moves forward. A fundamental issue is the inherent complexity and redundancy of the circadian system. The molecular clock consists of multiple interlocking feedback loops and built-in compensatory mechanisms as shown in Figure 1; if a drug perturbs one component, the system can sometimes adjust via alternate pathways to resist change. For example, a compound that stabilizes CRY proteins might lengthen the circadian period, but the clock could counterbalance by changing the expression of other regulators (such as increasing REV-ERB or kinase activity) to preserve homeostasis. Consequently, a single-target drug might show diminishing returns or inconsistent effects across different tissues or conditions. Indeed, some REV-ERB and ROR ligands that were very potent in cell culture assays produced only modest changes in circadian rhythms when tested in whole animals, in part because the intact organism’s clock can buffer against moderate disruptions. Achieving a strong therapeutic effect might require using combinations of clock modulators (a multi-target approach) or administering higher drug doses, but higher doses in turn raise the risk of off-target effects.

Off-target effects remain a significant limitation in the development and interpretation of clock-modulating therapeutics. Many of the small molecules currently studied were discovered through phenotypic screens rather than target-first strategies, increasing the likelihood that their biological effects stem from interactions with unintended proteins. For instance, the widely used REV-ERB agonist SR9009 has been shown to influence metabolic and proliferative pathways even in cells lacking REV-ERBα/β, suggesting noncanonical targets at higher concentrations that complicate attribution of its phenotypes solely to clock modulation [52]. Similarly, casein kinase inhibitors—another class of circadian modulators—exemplify this issue. The early CK1 inhibitor IC261 was later found to disrupt tubulin polymerization, leading to cytotoxic effects independent of its circadian function [75]. Such pleiotropic activity poses interpretive challenges, particularly in cancer or metabolic studies where parallel signaling networks may contribute to observed phenotypes. To disentangle these effects, rigorous target deconvolution is essential, referring to the systematic identification of the direct molecular targets and binding partners responsible for a compound’s observed biological activity following phenotypic screening. This process is particularly critical for circadian modulators discovered through unbiased approaches, where primary targets are often unknown.

Several complementary quantitative proteomics strategies are commonly employed for this purpose. These include thermal stability-based approaches such as the cellular thermal shift assay (CETSA), which detects ligand-induced stabilization of proteins in intact cells, and drug affinity responsive target stability (DARTS), which measures protection of target proteins from proteolytic degradation upon compound binding. In addition, mass spectrometry-based chemoproteomics techniques, including affinity-based pull-down assays and thermal proteome profiling, enable unbiased identification of drug–protein interactions across the proteome. Together, these methods provide powerful tools to confirm on-target engagement and distinguish primary mechanisms from off-target effects.

Techniques such as quantitative proteomics (e.g., thermal shift or drug affinity responsive target stability assays), CRISPR-mediated gene knockout, and orthogonal chemical probes should be used to confirm whether biological outcomes are truly mediated through engagement of circadian pathways. Without such validation, there is a risk of misattributing therapeutic benefits to clock modulation when they may arise from broader effects on cellular stress, metabolism, or cytoskeletal dynamics. Addressing these liabilities is critical for advancing clock-targeting compounds toward clinical relevance.

Another practical challenge lies in how we measure the effectiveness of a circadian drug in a clinical context. Unlike a medication such as an antibiotic where efficacy can be clearly seen in the elimination of an infection, a circadian modulator’s benefits may be more subtle and complex to quantify. What metric do we use to declare success? Is it the ability to reliably shift a patient’s internal clock by a certain amount, an increase in the amplitude of a particular hormonal rhythm, or improved sleep quality or energy levels? These kinds of endpoints require specialized monitoring tools, such as wrist actigraphy to track rest–activity cycles, assays for dim-light melatonin onset (to gauge internal phase), or determining internal circadian time through tissue samples [76,77,78]. Moreover, there is considerable individual variability in human circadian characteristics. People have different baseline phases and chronotypes (morning lark vs. night owl tendencies), so administering a phase-shifting drug at a suboptimal time of day could inadvertently worsen a person’s circadian alignment instead of improving it. All this points to a need for personalized chronotherapy, where not just the drug but the timing of its administration is tailored to the individual. The same drug given in the morning versus the evening might have a wide range of effects [8]. Therefore, determining the optimal dosing schedule (the “when” in addition to the “what” and “how much”) will be as important as choosing the right compound. This requirement adds complexity to clinical trials as well: studies may need to categorize participants by their chronotype or enforce specific dosing times, which can be logistically demanding.

In the specific context of cancer, the heterogeneity of tumors introduces additional hurdles. Not every tumor has the same circadian profile; some cancers might essentially be “clock-null” (having mutations or deletions in core clock genes) and thus could be unresponsive to a clock modulator that assumes a functioning clock pathway. For example, if a tumor has lost CRY or BMAL1 expression, administering a CRY-stabilizing compound or a REV-ERB agonist may do little to the cancer cells (even though it might still affect the patient’s normal tissues). Conversely, a tumor that lacks its own internal clock might, in theory, be more susceptible to influence by the host’s circadian environment, or such tumors might exploit a disrupted host clock to their advantage. Therefore, assessing a tumor’s circadian status could become an important step in deciding on therapy, for instance, by checking a biopsy for clock gene expression levels [7,54]. If a tumor is circadian-competent (i.e., its clock is intact and functional), then using a drug like SR9009 to hit that clock might be particularly effective. If a tumor is circadian-deficient, the strategy might shift toward utilizing chronotherapy (timing of other treatments) rather than relying on a direct clock modulator. This consideration adds another layer of complication to treating tumors, making personalized treatment plans even more challenging.

Another key challenge in translating circadian-targeting strategies lies in the context-dependent roles that clock mechanisms play across diseases. In oncology, for instance, certain tumors exhibit clock gene suppression as a driver of proliferation, making circadian disruption or clock inhibition a potentially beneficial therapeutic approach. Conversely, in aging and metabolic disorders, dampened rhythms often reflect systemic decline, and enhancing circadian amplitude may restore homeostatic functions. This dichotomy highlights the need for disease-specific strategies: clock activation may promote tissue resilience in neurodegeneration or metabolic syndrome, while selective clock inhibition may be more appropriate in rapidly proliferating cancers [6,39]. Moreover, targets such as REV-ERBs, CK1δ/ε, and CRY proteins represent mechanistically distinct nodes, each with varying degrees of tissue specificity and therapeutic reversibility. Future development pathways will require robust chronotyping tools, mechanistic understanding of the target cancer model, and a clearer understanding of how circadian interventions interact with disease heterogeneity at the molecular, cellular, and systemic levels.

For anti-aging interventions, another limitation is the inherently long timeline required to observe meaningful outcomes. Clinical trials aiming to extend healthspan or lifespan might need to run for many years to detect significant benefits, and it can be challenging to isolate the specific impact of a clock-modulating drug amidst the myriad other factors that influence aging (such as diet, physical activity, and concurrent health issues). Additionally, many older individuals are already on multiple medications, so introducing a clock-modulating drug could potentially lead to drug–drug interactions. This is especially challenging because the activity of various drug-metabolizing enzymes follows circadian patterns, meaning that a circadian modulator could alter the pharmacokinetics of other drugs, affecting their efficacy or side-effect profiles.

While preclinical studies have established compelling roles for circadian modulation in cancer suppression and metabolic regulation, translating these findings into safe and effective human therapies remains a significant challenge. Most current data derive from in vitro systems or animal models, which do not fully capture the complexity of human circadian physiology or inter-individual variability. Long-term safety of clock-targeting agents has not yet been established, particularly given the central role of circadian rhythms in diverse organ systems. Systemic manipulation of the clock may have unintended consequences in tissues with distinct oscillatory dynamics or circadian vulnerabilities. Moreover, clinical application is hindered by the difficulty of accurately monitoring circadian phase and amplitude in real-world settings, and by the lack of validated biomarkers for patient stratification. As such, while clock-modulating compounds offer intriguing therapeutic potential, a more nuanced understanding of their safety, specificity, and physiological context will be essential before they can be translated into viable clinical interventions. Translating findings from model organisms to humans is an overarching challenge. Mice, for example, are nocturnal and have much faster metabolic rates; a drug that strengthens circadian rhythms in mice might not have the same effect in diurnal humans, or it could even produce opposite outcomes due to fundamental species differences in physiology. The dosages and timing schedules that work in rodents often need significant adjustment for humans, who have slightly longer intrinsic circadian periods (around 24.2 h on average) and very different patterns of light exposure and behavior. Moreover, human genetic diversity means that natural variations (polymorphisms) in clock genes might cause individuals to respond in unique ways to a given clock modulator. Personalized factors could influence how a treatment affects someone’s clock by a lot, a little, or not at all.

In summary, although the idea of treating diseases by adjusting the circadian clock is very promising, it comes with distinct challenges that must be addressed. We will need to ensure that drugs are specific in their action (to minimize off-target effects), figure out the best timing regimens for dosing, closely monitor effects across different organ systems, and customize approaches to individual patients’ circadian profiles. As our understanding of circadian biology continues to grow and as new technologies emerge (including wearable devices for real-time rhythm monitoring, developing assays to determine internal body time, etc.), we will be better positioned to safely and effectively harness circadian modulators for medical therapy.

## 5. Future Directions

**Figure 4:**
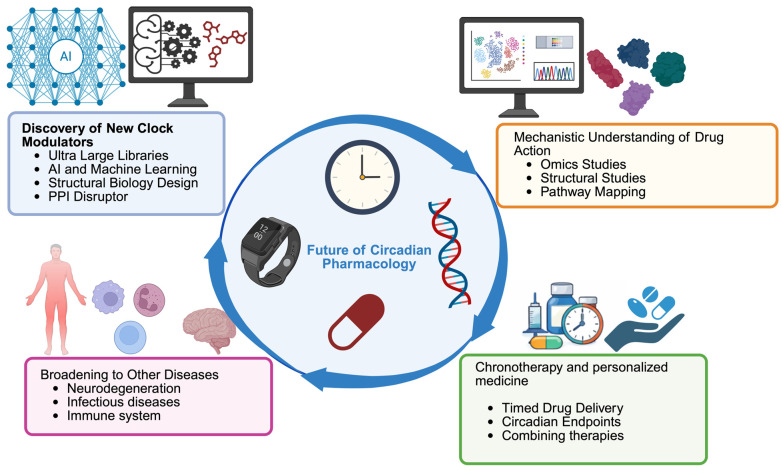
Future directions in circadian pharmacology. This figure highlights four major avenues shaping the next generation of circadian clock-targeted therapies. (1) Discovery of new clock modulators: Advances in ultra-large chemical libraries, artificial intelligence, machine learning, structural biology, and protein–protein interaction (PPI) disruptor design are expected to accelerate the identification of novel compounds acting on core clock components. (2) Mechanistic understanding of drug action: Multi-omics approaches, high-resolution structural studies, and pathway-level analyses will help clarify how clock-modulating compounds influence downstream networks. (3) Chronotherapy in clinical practice: Optimizing the timing of drug administration, incorporating circadian biomarkers into trials, and combining clock modulators with standard therapies represent key steps toward more effective and time-aligned medical interventions. (4) Broadening to other diseases: Beyond cancer and aging, circadian-based interventions have potential applications in neurodegeneration, infectious diseases, and immune-related disorders, reflecting the pervasive influence of circadian timing across physiology and pathology.

The interface of circadian biology with pharmacology is a rapidly evolving frontier, and the coming years are likely to witness significant advances in both science and therapeutic application of clock modulators. Below are several key directions and emerging concepts that are shaping the future of this field, summarized in Figure 4.

1. Discovery of New Clock Modulators: While the list of known circadian-modulating compounds has grown considerably, it is still far from complete. Future research will likely involve screening much larger and more diverse libraries of chemicals including previously unexplored natural product collections and vast virtual libraries to uncover novel chemotypes that influence the clock. Tools like machine learning may augment high-throughput phenotypic screens [79], helping predict which chemical structures are likely to lengthen or shorten the circadian period before synthesis or testing. Fragment-based drug discovery could also be applied to core clock proteins (such as cryptochrome or the BMAL1:CLOCK complex), where small binding fragments are identified and then chemically expanded into potent modulators. We also expect continued targeted searches for compounds that affect clock-regulating enzymes, like specific kinases or phosphatases, since post-translational modifications are pivotal in clock control. Structural biology breakthroughs, such as detailed maps of key clock protein interfaces like the CRY–FBXL3 binding interface [80] and the tertiary structure of the CLOCK:BMAL1 complex [81] and detailed CLOCK transactivation domain alanine mutation scanning [35], provide templates for rational drug design efforts. Consequently, the next generation of clock-targeting drugs might include more agents that disrupt protein–protein interactions (for instance, a molecule preventing binding of CLOCK proteins on tandem E-boxes) [35].
2. Mechanistic Understanding of Clock Drug Effects: As new clock-modulating compounds are discovered, it will be crucial to delineate exactly how they produce their effects at a molecular and cellular level. Future studies will likely use systems biology and various “omics” techniques to map the ripple effects of clock modulation across cellular networks. For example, comparing the transcriptome and metabolome of cells treated with a clock enhancer versus a clock inhibitor could highlight which downstream pathways (such as those governing metabolism or DNA repair) are shifted, helping to explain observed therapeutic outcomes. In parallel, detailed structure–function analyses like X-ray crystallography or cryo-electron microscopy of clock proteins bound to these modulators will inform efforts to optimize drug potency and selectivity.
3. Chronotherapy in Clinical Trials: An important future direction is the incorporation of circadian principles into the design of clinical trials. We expect to see more studies where the timing of treatment is a key variable, for instance, administering cancer drugs at specific circadian phases to maximize their toxicity to tumors while minimizing harm to healthy tissue. Furthermore, upcoming early-phase trials of dedicated clock modulators will likely include circadian rhythm measurements as part of their endpoints.
4. Personalized Chronomedicine: Because circadian characteristics vary widely from person to person (differences in chronotype, intrinsic period, amplitude of rhythms, etc.), personalization is likely to be a key element in the future of circadian therapeutics. Assessment of patients’ circadian profile using wearable rhythm trackers, melatonin assays, or the bodytime assay is going to be essential in tailoring treatments.
5. Broadening to Other Diseases: Although we have concentrated on cancer and aging, circadian modulators have potential applications across a broad spectrum of diseases. Many chronic inflammatory disorders, such as rheumatoid arthritis, show circadian patterns in symptoms (e.g., early morning joint stiffness and peaks in inflammatory activity), suggesting that clock-targeted drugs might help blunt these daily symptom cycles. Likewise, neurodegenerative diseases commonly involve sleep disturbances and altered clock gene expression; boosting circadian function might slow neurodegeneration. In principle, any disease influenced by timing or by rhythmic gene expression could become a target for chronotherapy.

In conclusion, the future looks promising for the field of circadian pharmacology. The idea of “circadian medicine” is rapidly evolving from a theoretical possibility to a tangible approach in healthcare. If the current trajectory of discovery and innovation continues, we could soon have novel therapies that not only enhance patients’ quality of life by reinstating robust daily rhythms but also simultaneously combat major diseases such as cancer, metabolic syndrome, or neurodegenerative disorders. Similarly, strengthening our internal timekeeping system might prove to be a critical component of promoting healthier aging and extending longevity. The coming years of research and clinical exploration will put these exciting possibilities to the test and may very well usher in an era of medicine where, quite literally, timing is everything.

## Data Availability

No new data were generated from this work.
